# Population Expansion and Genetic Structure in *Carcharhinus* *brevipinna* in the Southern Indo-Pacific

**DOI:** 10.1371/journal.pone.0075169

**Published:** 2013-09-25

**Authors:** Pascal T. Geraghty, Jane E. Williamson, William G. Macbeth, Sabine P. Wintner, Alastair V. Harry, Jennifer R. Ovenden, Michael R. Gillings

**Affiliations:** 1 Department of Biological Sciences, Macquarie University, Sydney, New South Wales, Australia; 2 Cronulla Fisheries Research Centre of Excellence, New South Wales Department of Primary Industries, Sydney, New South Wales, Australia; 3 KwaZulu-Natal Sharks Board, Umhlanga Rocks, KwaZulu-Natal, South Africa; 4 Centre for Sustainable Tropical Fisheries and Aquaculture, James Cook University, Townsville, Queensland, Australia; 5 Molecular Fisheries Laboratory, the University of Queensland, St. Lucia, Queensland, Australia; University of Lausanne, Switzerland

## Abstract

**Background:**

Quantifying genetic diversity and metapopulation structure provides insights into the evolutionary history of a species and helps develop appropriate management strategies. We provide the first assessment of genetic structure in spinner sharks (*Carcharhinus brevipinna*), a large cosmopolitan carcharhinid, sampled from eastern and northern Australia and South Africa.

**Methods and Findings:**

Sequencing of the mitochondrial DNA NADH dehydrogenase subunit 4 gene for 430 individuals revealed 37 haplotypes and moderately high haplotype diversity (*h* = 0.6770 ±0.025). While two metrics of genetic divergence (Φ_ST_ and *F*_ST_) revealed somewhat different results, subdivision was detected between South Africa and all Australian locations (pairwise Φ_ST_, range 0.02717–0.03508, *p* values ≤ 0.0013; pairwise *F*_ST_ South Africa vs New South Wales = 0.04056, *p* = 0.0008). Evidence for fine-scale genetic structuring was also detected along Australia’s east coast (pairwise Φ_ST_ = 0.01328, *p* < 0.015), and between south-eastern and northern locations (pairwise Φ_ST_ = 0.00669, *p* < 0.04).

**Conclusions:**

The Indian Ocean represents a robust barrier to contemporary gene flow in *C*. *brevipinna* between Australia and South Africa. Gene flow also appears restricted along a continuous continental margin in this species, with data tentatively suggesting the delineation of two management units within Australian waters. Further sampling, however, is required for a more robust evaluation of the latter finding. Evidence indicates that all sampled populations were shaped by a substantial demographic expansion event, with the resultant high genetic diversity being cause for optimism when considering conservation of this commercially-targeted species in the southern Indo-Pacific.

## Introduction

Patterns of genetic variability in extant taxa have been generated by events and processes occurring over evolutionary time scales. Genetic bottlenecks and demographic expansions, coupled with associated fluctuations in effective population size, are examples of such events, respectively manifesting as low and, eventually, high levels of genetic diversity [1-8]. Evolutionary processes that influence genetic variability, however, need not be characterised by pronounced reduction or elevation in diversity. In a range of taxa, barriers to dispersal and gene flow caused by geographic separation or long-term behavioural traits have led to spatial partitioning of genetic diversity. Cessation of gene flow results in spatial genetic differentiation [9-13], and ultimately, speciation due to natural selection, genetic drift and mutation [14-16]. Quantifying genetic diversity and metapopulation structure, therefore, can provide insight into the evolutionary history and behaviour of a species and, in turn, the most appropriate strategy for its management.

In the marine environment, generating accurate, representative estimates of genetic diversity and population structure can be challenging. Cryptic barriers to dispersal and inherent uncertainties pertaining to the spatial extent of gene flow within a species make the most informative experimental designs difficult to determine, notwithstanding the practical issues associated with the collection of highly-vagile marine taxa. For example, various members of the Carcharhinidae represent large, cosmopolitan shark species occupying predominantly continental-shelf waters [17]. Species such as the dusky ( *Carcharhinus* *obscurus* ), sandbar ( *Carcharhinus* *plumbeus* ), bull ( *Carcharhinus* *leucas* ) and common blacktip ( *Carcharhinus* *limbatus* ) shark are capable of travelling considerable distances, and are suspected to undertake long-range migrations [18-23]. These species are also dependent on shallow coastal habitats for birthing and offspring development [22,24-27], with mounting evidence demonstrating philopatric behaviour in juveniles and, more notably, in gravid females [12,28-31]. This trait suggests that, for some carcharhinid sharks, spatial genetic connectivity may be lower than otherwise predicted based on vagility and demonstrated patterns of movement. The contrast between long-range dispersal ability and the potential for sex-specific disruption of gene flow between geographically proximate locations provides a complex context within which to decipher genetic structure. Given the implications for management and conservation, however, this same dichotomy highlights the importance of an understanding of spatial genetic subdivision in shark species.

Genetic structure has been investigated in several carcharhinids at a range of geographic scales [32]. Studies on global phylogeography have consistently shown that large oceanic expanses are robust barriers to gene flow [33-38]. Genetic subdivision has also been documented over finer spatial scales and attributed to either philopatric behaviour or historic events causing geographic isolation [12,28,30,31,35,39-42].

The spinner shark ( *Carcharhinus* *brevipinna* ) has thus far been neglected in the population genetic literature. No research on genetic diversity or stock structure has been conducted in any part of its cosmopolitan range, which includes much of the world’s tropical and warm-temperate continental shelf waters [17]. *Carcharhinus* *brevipinna* is predominantly a by-catch or secondary target species, but is nevertheless an important component of commercial catches in multi-species shark fisheries around the world [43-50]. Furthermore, owing to confusion with the ‘blacktip’ shark, commercial catch records of *C* *. brevipinna* are most likely gross underestimates in some regions. Recreational catch rates are also suspected to be substantial, however, as for most shark species, they remain unquantified. In Australian waters, considerable numbers of *C* *. brevipinna* are landed along the eastern, northern and western coastlines where they are harvested using demersal longlines, demersal and pelagic gillnets, and handlines [51-55]. In eastern Australia, a fishery-observer study revealed this species to be the third most abundant large shark caught in the New South Wales Ocean Trap and Line Fishery (NSW OTLF) [53].

*Carcharhinus* *brevipinna* is a schooling species known to frequent nearshore waters as adults and utilise inshore nursery habitats as juveniles [24,56-59]. As such, *C* *. brevipinna* is considered highly vulnerable to fishing pressure and human-induced habitat alteration, and is hence globally IUCN listed as ‘near threatened’ [60]. Despite this, long-term catch-data sets have provided evidence for stock stability in *C* *. brevipinna* . Carlson et al. [50] proposed that growth overfishing had not occurred on this species in the heavily fished western North Atlantic, with the average landed size remaining stable from 1994–2009. Furthermore, the abundance of *C* *. brevipinna* in this fishery appears to have remained largely unchanged, with some evidence for increase over the same period [50]. Similar findings were reported by Dudley and Simpfendorfer [45] from the western Indian Ocean, who revealed stable catch per unit effort (CPUE) and stable/increasing size at capture from 1978–2003. Having experienced comparatively lower targeted-fishing pressure on a global scale, *C* *. brevipinna* has not been subject to the same concern or scrutiny regarding the status of its populations as that levelled at species such as *C* *. obscurus* and *C* *. plumbeus* [61-64]. However, the life-history characteristics of *C* *. brevipinna* suggest a similar vulnerability to overfishing and to slow intrinsic rates of population recovery [44,48,65-69]. Furthering our understanding of global *C* *. brevipinna* populations, therefore, may be considered prudent.

Here we assess genetic structure and diversity in *C* *. brevipinna* using mitochondrial DNA (mtDNA) sequence data. We test a null hypothesis of genetic homogeneity throughout Australian and South African waters, and discuss the evolutionary history of the species in the region. We generate an estimate of scientific-observer accuracy in identifying *C* *. brevipinna* in an eastern Australian large-shark fishery, and also discuss the implications of our findings for fisheries management and conservation.

## Materials and Methods

### Ethics Statement

Tissues were sampled from New South Wales (NSW) waters according to a protocol approved by the NSW Government Primary Industry (Fisheries) Animal Care and Ethics Research Authority (Permit ACEC REF 07/03 – CFC).

### Sample collection

**Figure 1:**
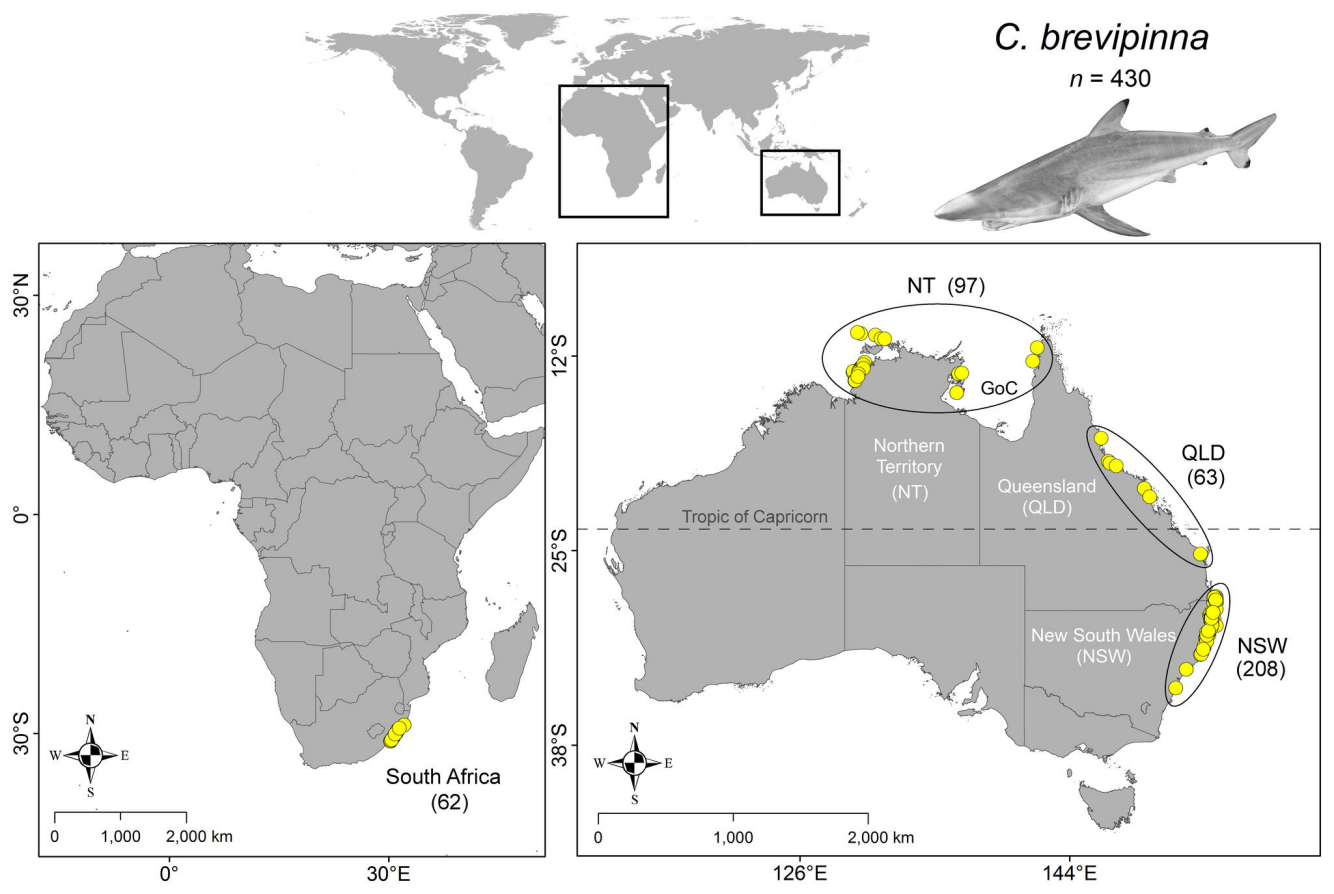
Collection locations for tissues included in genetic structure and diversity analyses. Sample numbers for each putative population are in parentheses. GoC = Gulf of Carpentaria.

**Figure 2:**
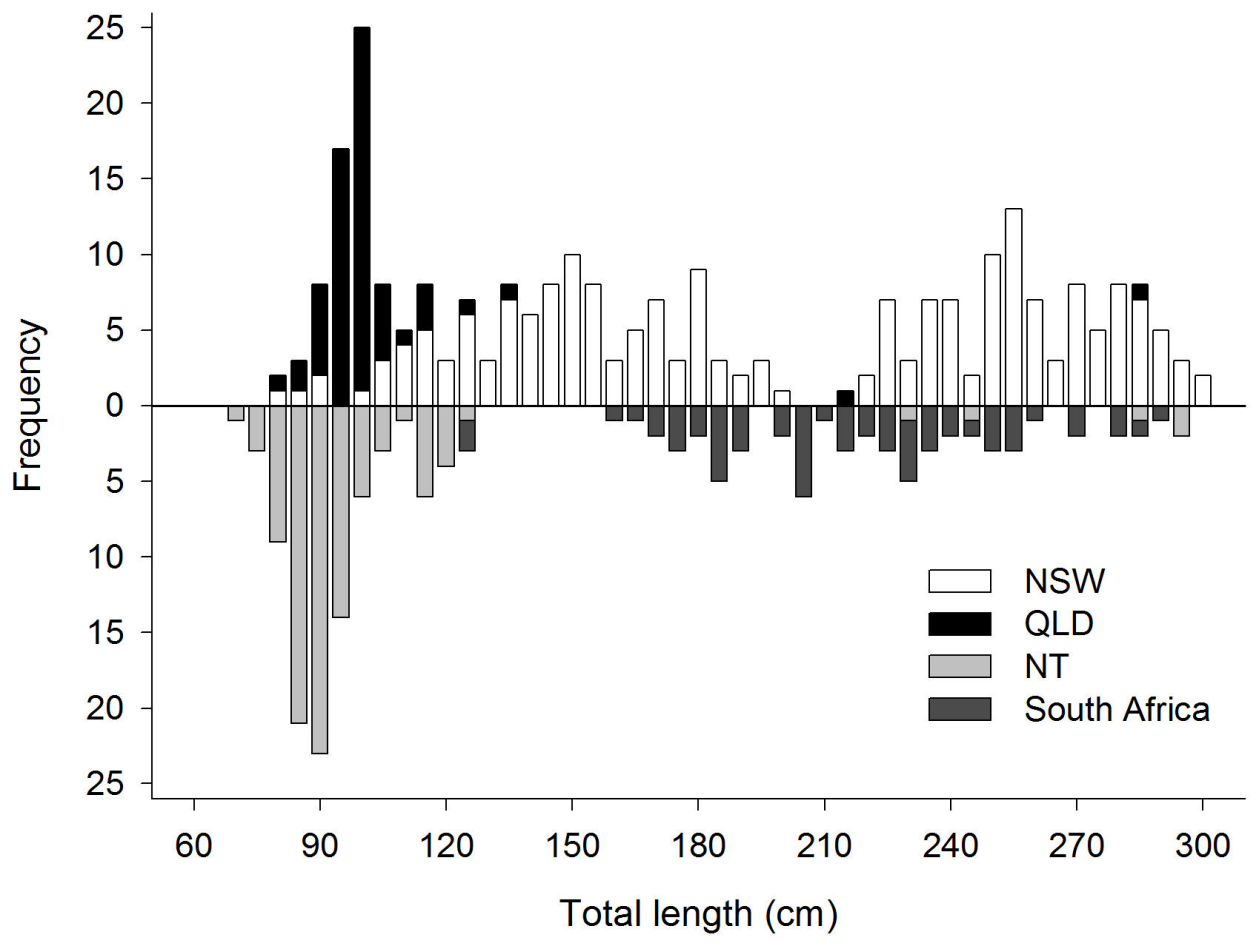
Length-frequency distribution of individuals from which tissues were sampled.

Shark tissues were collected from a range of locations in the southern Indo-Pacific (Figure 1) using a variety of fishery-dependent methods. From NSW waters, tissues were harvested during 2007–2010 from landed catch by scientific observers on-board commercial shark-fishing vessels within the NSW OTLF. These samples were taken from individuals spanning the entire size range of the species (Figure 2). A small quantity (<2 g) of white muscle tissue was excised from each specimen, immediately preserved in 95% reagent grade ethanol, and stored at room temperature. Additional samples, collected during 2000–2010, were obtained from more distant locations, including from the waters of north-western Northern Territory (NT), Gulf of Carpentaria (GoC) and Queensland (QLD) in northern Australia, as well as from the east coast of South Africa (Figure 1). Tissues from north-western NT, GoC and QLD were sampled from predominantly neonate and small-juvenile individuals from landed catch by observers within their respective commercial shark fisheries (Figure 2), and were preserved in 20% dimethylsulphoxide (DMSO) solution. Fin-clip samples from South Africa, preserved in 100% ethanol, were collected from adult and sub-adult sharks caught in the Kwazulu-Natal beach protection nets (Figure 2). For South African specimens, pre-caudal length (PCL) measurements were converted to total length (TL) using the morphometric equation published in Allen and Wintner [67]. Additional samples were obtained from QLD and NSW waters by sampling sharks caught in government bather protection programs [59,70].

### DNA extraction, amplification and sequencing

To obtain mtDNA sequence data, total genomic DNA was extracted from 5 mg of tissue using a modified salting-out protocol [71]. Samples were digested with 10 µl of Proteinase-K (10 mg·ml^-1^) in 580 µl of TNES [50 mM Tris.HCl (pH 7.5), 400 mM NaCl, 20 mM EDTA and 0.5% SDS] by incubation overnight at 55 °C. Proteins were precipitated by adding 170 µl of 5 M NaCl followed by microcentrifugation at 14,000 rpm for 5 min. Supernatant (600 µl) was recovered into a fresh tube and the DNA precipitated by adding 600 µl of ice-cold 100% ethanol. Tubes were stored at −20 °C for approximately 1 h. DNA was then recovered by microcentrifugation at 14,000 rpm for 15 min, and the ethanol decanted. The resulting DNA pellet was washed with 200 µl of 70% ethanol, 100 mM sodium acetate solution, and microcentrifuged at 14,000 rpm for 3 min. Following decanting, all remaining ethanol was removed using a micropipette. DNA was air-dried, resuspended in 100 µl of TE buffer [10 mM Tris.HCl (pH 7.6) and 1 mM EDTA] and stored at −20 °C. DNA yield was checked on a 1.0% agarose TBE (90 mM TRIS-borate and 2 mM EDTA) gel run at 110 V.

Polymerase Chain Reaction (PCR) was used to amplify the mitochondrial DNA NADH dehydrogenase subunit 4 (ND4) gene from all tissue samples. Reactions were carried out in 50 µl volumes containing 1 µl of DNA template, 1× GoTaq Colourless reaction buffer [consisting of 1.5 mM MgCl_2_ and 200 µM deoxynucleoside triphosphates (dNTPs)] (Promega, Madison, WI, USA), 0.5 µl of RNase (1 mg·ml^-1^), and 0.5 µM of each of the primers ND4 (5’ CAC CTA TGA CTA CCA AAA GCT CAT GTA GAA GC) [72] and H12293-LEU (5’ TTG CAC CAA GAG TTT TTG GTT CCT AAG ACC) [73]. Amplifications were performed in an Eppendorf ep gradient S Mastercycler (Eppendorf AG, Hamburg, Germany), using thermal cycling conditions consisting of an initial denaturation (94 °C for 3 min), followed by 35 cycles of 94 °C for 15 s, 60 °C for 30 s and 72 °C for 1 min, with a final extension step of 72 °C for 10 min, and soak/finish at 4 °C. PCR products were visualised on a 2.0% agarose TBE gel, run at 110 V, and stained with GelRed (Biotium Inc., Hayward, CA, USA). PCR products were purified prior to sequencing using Exosap-IT (USB Corporation distributed by GE Healthcare Bio-Sciences, Rydalmere, Australia). Sequencing was performed with an Applied Biosystems 3130xl Genetic Analyzer 16-array capillary sequencer (Life Technologies, Carlsbad, CA, USA). Sequencing reactions and analyses were carried out by the Macquarie University (MQ) DNA Sequencing Facility using Big Dye Terminator reactions and the forward PCR primer only.

### Sequence alignment and ID validation

Sequences were trimmed and edited manually. Edited sequences were entered into Biomanager (https://biomanager.info) and aligned using the ClustalW (accurate) algorithm [74]. GenBank reference sequences for *C* *. brevipinna* were available for the cytochrome oxidase I (CO1) gene, but not for ND4, prior to this study. Therefore, to validate that the study species had been correctly identified and also to determine the species identity of any misidentified individuals, randomly-selected representatives from each separate haplotype determined from the alignment output were amplified for the CO1 gene using the primers Fish F1 (5’ TCA ACC AAC CAC AAA GAC ATT GGC AC) and Fish R1 (5’ TAG ACT TCT GGG TGG CCA AAG AAT CA) [75]. PCRs were carried out as above, with thermal cycling conditions consisting of an initial denaturation (95 °C for 5 min), followed by 30 cycles of 95 °C for 15 s, 55 °C for 30 s and 72 °C for 1 min, with a final extension step of 72 °C for 7 min, and soak/finish at 4 °C. PCR products were purified and sequenced following the same protocol outlined for the ND4 locus. Resultant CO1 sequences were compared to reference sequences in Genbank for species recognition.

### ND4 sequence analysis

To identify and characterise mitochondrial haplotypes, aligned *C* *. brevipinna* ND4 sequences were imported to Arlequin 3.5.1.2 [76]. A sequence representing each haplotype was lodged in GenBank (Accession codes KF612545-KF612581). The frequency of, and mutational steps between, haplotypes were assessed by generating statistical parsimony haplotype networks in TCS 1.21 using the default settings [77]. Phylogenetic relationships among haplotypes were inferred using the maximum likelihood method based on the Tamura-Nei model [78], and generated in MEGA 5 [79] with 1,000 bootstrap replicates. The best-fitting model of nucleotide substitution, as offered by MEGA 5, was determined by likelihood ratio tests and calculations of Akaike and Bayesian Information Criteria performed in jModelTest 2.1.1 [80]. To assess the ability of the ND4 region to differentiate between carcharhinids, the phylogram was rooted with a range of morphologically similar species, as well as with two sphyrnid species as outgroups.

Genetic diversity indices were obtained with Arlequin using the Tamura-Nei substitution model [78], and included polymorphism statistics, number of haplotypes, haplotype diversity (h) and nucleotide diversity (*π*). Harpending’s raggedness index (*H*_*RI*_) was estimated from nucleotide mismatch distributions constructed in Arlequin under the sudden demographic expansion model with 20,000 bootstrap replicates [81]. Tajima’s *D* and Fu’s *F* neutrality indices were also estimated in Arlequin, and are indicative of departures from mutation-drift equilibrium or patterns of selection [82,83]. In conjunction with *H*_*RI*_, the latter two analyses can be used to determine if a population has undergone an expansion event (possibly following a genetic bottleneck). Mismatch distributions will be multi-modal (or ragged) in a stable population, where the generation of new mutations is offset by random drift, and uni-modal for expanding populations, where new mutations accumulate faster than their loss due to drift [81]. For Tajima’s *D* and Fu’s *F*, signals of population expansion are denoted by significant negative test statistic values. Statistical significance was assessed here, following 20,000 simulated samples, at α = 0.05 and α = 0.02 for *D* and *F* values respectively [83].

### Population genetic structure

To test the null hypothesis of panmixia (genetic homogeneity) in Australian and South African waters for *C* *. brevipinna* , an analysis of molecular variance (AMOVA) [84] was implemented in Arlequin to evaluate the overall extent of net genetic subdivision between sample locations. We employed two *F*-statistic metrics of genetic divergence: Φ_ST_ [84] and *F*_ST_ [85]. While Φ_ST_ has been regarded as the superior metric on the basis of its incorporation of a measure of genetic distance between haplotypes, frequency-based *F*_ST_ has been proposed as potentially a more appropriate measure of genetic differentiation among locations where migration is theoretically occurring at a faster rate than mutation [86]. Φ_ST_ was calculated via the computing of a distance matrix using the Tamura-Nei model [78] for estimation of genetic distance between sequences, while *F*_ST_ used haplotype frequencies only. AMOVA partitioned genetic variance among, and within, sample locations, and calculated overall Φ_ST_ and *F*_ST_ fixation indices. Genetic differentiation between each pair of locations was also measured by calculating pairwise Φ_ST_ and *F*_ST_ estimates. Statistical significance was determined following 20,000 permutations of the sequence data and, in the case of pairwise Φ_ST_ and *F*_ST_, assessed at an initial critical significance level of α = 0.0083 (adjusted from α = 0.05) following sequential Bonferroni correction for six simultaneous comparisons [87,88]. The AMOVA structure consisted of one group made up of the following four putative populations: NSW (*n* = 208), QLD (*n* = 63), NT (*n* = 97) and South Africa (*n* = 62) (Figure 1). The analysis outlined above is henceforth referred to as the ‘original analysis’. Prior to conducting this large-scale AMOVA, we investigated the extent of genetic subdivision on a finer scale between GoC (*n* = 43) and north-western NT (*n* = 54) waters. This analysis indicated genetic homogeneity (fixation indices: Φ_ST_ = 0.00035, *p* > 0.39; *F*_ST_ = 0.00151, *p* > 0.31), hence providing justification for pooling GoC and north-western NT samples to create one northern population termed ‘NT’.

*Carcharhinus* *brevipinna* sample sizes were clearly biased towards NSW (Figure 1), where 208 samples were collected compared to 62, 63 and 97 samples from the other three locations. We evaluated the influence of this sampling bias on the *F*-statistics of pairwise population comparisons involving NSW via random re-sampling simulations. Ten thousand replicate random sample-sets of *n* = 60 (for comparison with QLD and South Africa, but not NT owing to its larger original sample size), *n* = 100 and *n* = 150 were selected without replacement from the NSW population, while QLD, NT and South African sample sizes were kept unchanged. Population pairwise Φ_ST_ and associated *p* values were generated for each replicate random sample-set in Arlequin using the batch processing function and permutation settings as outlined above. Resultant Φ_ST_ and *p* value distributions were plotted, and the likelihood of producing a result contradictory to that of the original analysis was calculated as either the proportion of *p* values ≤ 0.05 or > 0.05, depending on the result of the original analysis. That is, if the original pairwise *p* value was significant (*p* ≤ 0.05), the likelihood of a contradictory result equals the absolute number of *p* values > 0.05/10,000.

The ‘Isolation by Distance’ (IBD) hypothesis was also tested to determine if inter-population genetic distances increased linearly with geographic distance. Genetic (Φ_ST_) and geographic (km, by sea) distances between the four putative populations were calculated in GenAlEx [89] and ArcMap 10.0 (ESRI), respectively. Pairwise genetic and geographic distance matrices were correlated using a Mantel test, with a test for a significant relationship by 9,999 random permutations, also implemented in GenAlEx.

### Rarefaction analysis

To determine whether sample sizes adequately represented population genetic variability, rarefaction exact curves were generated to qualitatively assess the proportion of haplotypic diversity sampled at each of the four locations. The expected number of haplotypes found for a given sample number (from one to the total sample size obtained at each location) was calculated using the rarefaction formula of Hurlbert [90], and executed in the statistical package R [91]. A trend towards an asymptotic relationship infers haplotype saturation, i.e. that the majority of the available genetic diversity was likely sampled at that location and that more intensive sampling is likely to yield few additional haplotypes. In contrast, a steep slope suggests that a large fraction of the available haplotype diversity remains unsampled.

## Results

### Fishery-observer accuracy in NSW waters

**Figure 3:**
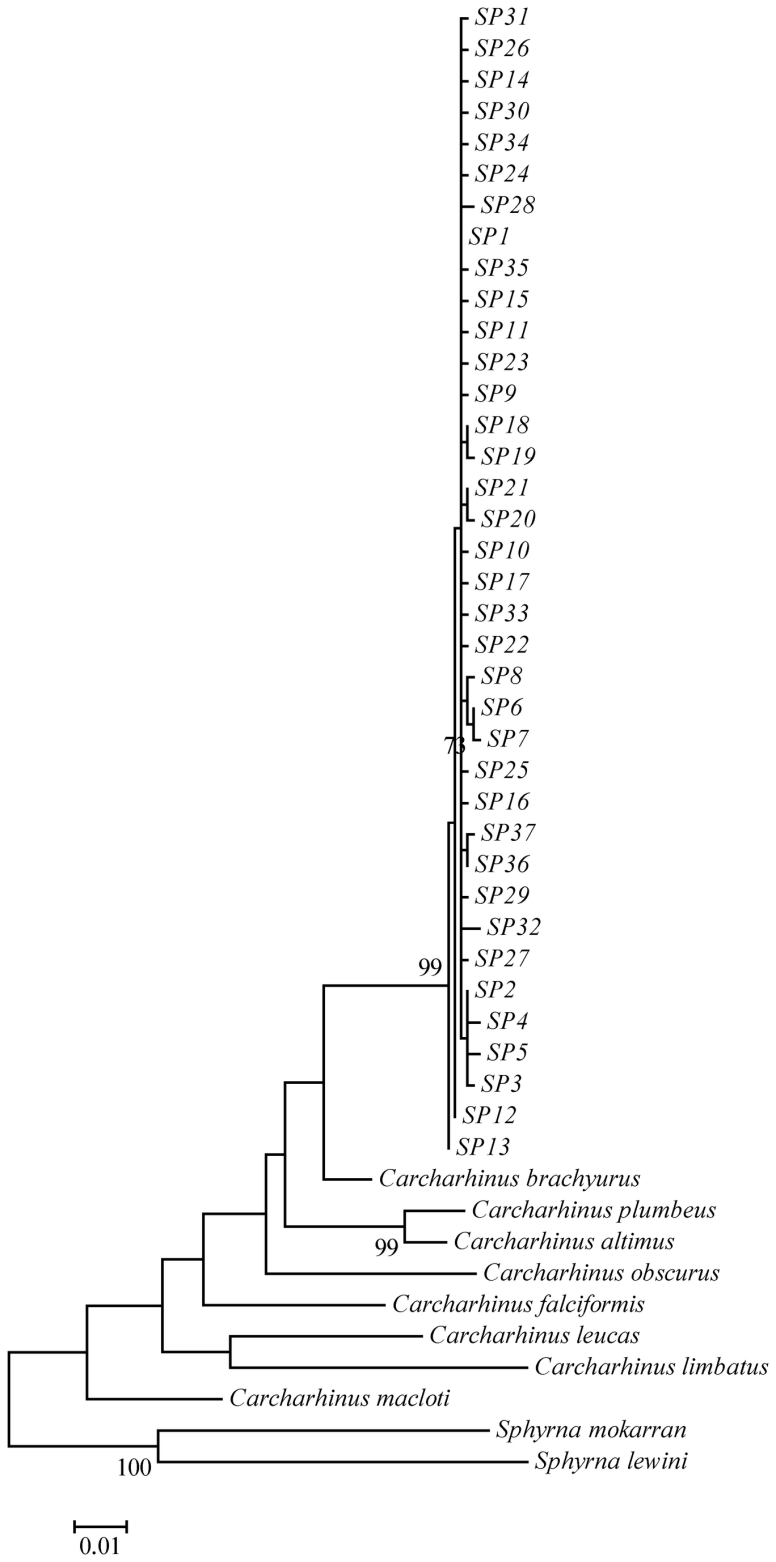
Maximum likelihood phylogenetic tree for *Carcharhinus* *brevipinna* haplotypes. Nodal bootstrap support is displayed where ≥ 70%. Scale represents the proportion of polymorphic sites between haplotypes.

The ND4 gene region proved to be capable of distinguishing a range of morphologically-similar carcharhinids (Figure 3), as previously shown by Tillett et al. [55]. Genetic validation was possible for a total of 190 sharks identified by scientific observers as *C* *. brevipinna* in the NSW OTLF from 2007–2010. Of these, 187 were genetically confirmed to be *C* *. brevipinna* , translating to an observer-accuracy estimate of 98.4% for the identification of this species in the fishery (Table 1). Misidentified individuals (*n* = 3) comprised two *C* *. limbatus* and one *C* *. obscurus* (Table 1).

**Table 1:** Fishery-observer identification accuracy.

Genetic identification	Observer identified *C* *. brevipinna* (*n* = 190)
*C* *. brevipinna*	98.4 (187)
*C* *. limbatus*	1.1 (2)
*C* *. obscurus*	0.5 (1)

Percentage (individual counts in parentheses) of each genetically-identified shark species from observer-identified *Carcharhinus* *brevipinna* in the New South Wales Ocean Trap and Line Fishery.

### Genetic diversity and summary statistics

An 857 base pair mtDNA ND4 sequence was obtained for 430 *C* *. brevipinna* individuals collected from Australian and South African waters (Figure 1). A total of 37 haplotypes were defined, characterised by 41 polymorphic sites composed of 40 transitions and one transversion (Table S1). A phylogenetic tree placed all haplotypes into a single, shallow clade (Figure 3). One haplotype (SP1) clearly dominated the sample set, and was found in all four populations in reasonably similar proportions (Table 2). The same number of haplotypes (*n* = 23) was found in NSW and NT waters, despite NSW having over double the sample size (Table 3). NSW exhibited six haplotypes endemic to the area, whereas NT displayed five. Almost identical sample sizes revealed 17 haplotypes from QLD waters and 11 from South African waters, with two unique haplotypes defined from each location (Table 3). Haplotype (h) and nucleotide (*π*) diversities were very similar, and high in the case of the former and low in the case of the latter, across three of the four putative populations (QLD, NT and South Africa; *h*, range 0.7279–0.7493; π, range 0.0015–0.0016) (Table 3). Comparatively lower diversity was observed in NSW waters (*h* = 0.5984, π = 0.0010). All mismatch distributions were consistent with the sudden population expansion model, with no significant deviation from a uni-modal distribution (*H*_*RI*_, range 0.054–0.099) (Table 3). In support of this, all four putative populations displayed significant negative neutrality indices (*D*, range -2.245 – -1.506; *F*, range -23.626 – -4.464) (Table 3).

**Table 2:** Haplotype relative frequencies observed from each sampling location.

	Relative frequency				
Haplotype	NSW (*n* = 208)	QLD (*n* = 63)	NT (*n* = 97)	South Africa (*n* = 62)	GenBank Accession
*SP1*	0.625	0.492	0.505	0.468	KF612545
*SP2*	0.082	0.111	0.124	0.065	KF612546
*SP3*	0.005	0.032	0.010	–	KF612547
*SP4*	0.010	0.063	0.041	0.016	KF612548
*SP5*	–	–	0.010	–	KF612549
*SP6*	0.019	–	0.010	–	KF612550
*SP7*	–	–	–	0.065	KF612551
*SP8*	0.005	0.016	0.031	–	KF612552
*SP9*	–	–	–	0.032	KF612553
*SP10*	–	0.016	–	0.145	KF612554
*SP11*	–	–	0.010	0.016	KF612555
*SP12*	–	–	0.021	0.048	KF612556
*SP13*	–	–	0.010	0.016	KF612557
*SP14*	–	–	0.010	–	KF612558
*SP15*	–	–	0.010	–	KF612559
*SP16*	–	–	0.010	–	KF612560
*SP17*	0.019	0.016	0.021	0.032	KF612561
*SP18*	0.053	0.048	0.021	–	KF612562
*SP19*	–	–	0.010	–	KF612563
*SP20*	–	0.016	0.010	–	KF612564
*SP21*	0.038	–	0.041	–	KF612565
*SP22*	0.024	0.063	0.021	–	KF612566
*SP23*	0.005	–	0.010	–	KF612567
*SP24*	0.005	–	0.010	–	KF612568
*SP25*	–	0.016	–	–	KF612569
*SP26*	–	0.016	–	–	KF612570
*SP27*	0.005	0.016	–	–	KF612571
*SP28*	0.005	0.016	–	–	KF612572
*SP29*	0.019	0.016	0.010	–	KF612573
*SP30*	0.010	0.016	–	–	KF612574
*SP31*	0.010	–	–	–	KF612575
*SP32*	0.010	–	–	–	KF612576
*SP33*	0.014	–	–	–	KF612577
*SP34*	0.005	–	–	–	KF612578
*SP35*	0.005	–	–	–	KF612579
*SP36*	0.019	0.032	0.041	0.097	KF612580
*SP37*	0.010	–	–	–	KF612581

SP1-37 = Observed *Carcharhinus* *brevipinna* mitochondrial DNA ND4 haplotypes.

**Table 3:** Genetic diversity indices observed for *Carcharhinus* *brevipinna* sample locations in the southern Indo-Pacific.

Location	*n * ^*a*^	*n*_*H*_ ** ^*b*^	*n*_*Hq*_ ** ^*c*^	*h * ^*d*^	*π * ^*e*^	*H*_*RI*_^*f*^	*D * ^*g*^	*F * ^*h*^
NSW	208	23	6	0.5984 (±0.040)	0.0010 (±0.0008)	0.074	− 2.245***	− 23.626***
QLD	63	17	2	0.7424 (±0.056)	0.0015 (±0.0011)	0.057	− 2.056**	− 13.080***
NT	97	23	5	0.7279 (±0.047)	0.0015 (±0.0010)	0.054	− 2.163**	− 22.072***
South Africa	62	11	2	0.7493 (±0.050)	0.0016 (±0.0011)	0.099	− 1.506*	− 4.464*
Pooled	430	37	•	0.6770 (±0.025)	0.0013 (±0.0009)	0.064	− 2.252***	− 29.294***

a Sample size (n ,

b number of haplotypes (*n*_*H*_),

c number of unique haplotypes (*n*_*Hq*_),

d haplotype diversity (h ,

e nucleotide diversity (*π*),

f Harpending’s raggedness index (*H*_*RI*_),

g Tajima’s (*D*) and ^h^ Fu’s (*F*) tests of selective neutrality, Values in parentheses represent standard deviations (s.d.).

• value not applicable.

* denotes significance at the *p* ≤ 0.05 level, **p ≤ 0.01, ***p ≤ 0.001.

### Rarefaction and optimum sample size

**Figure 4:**
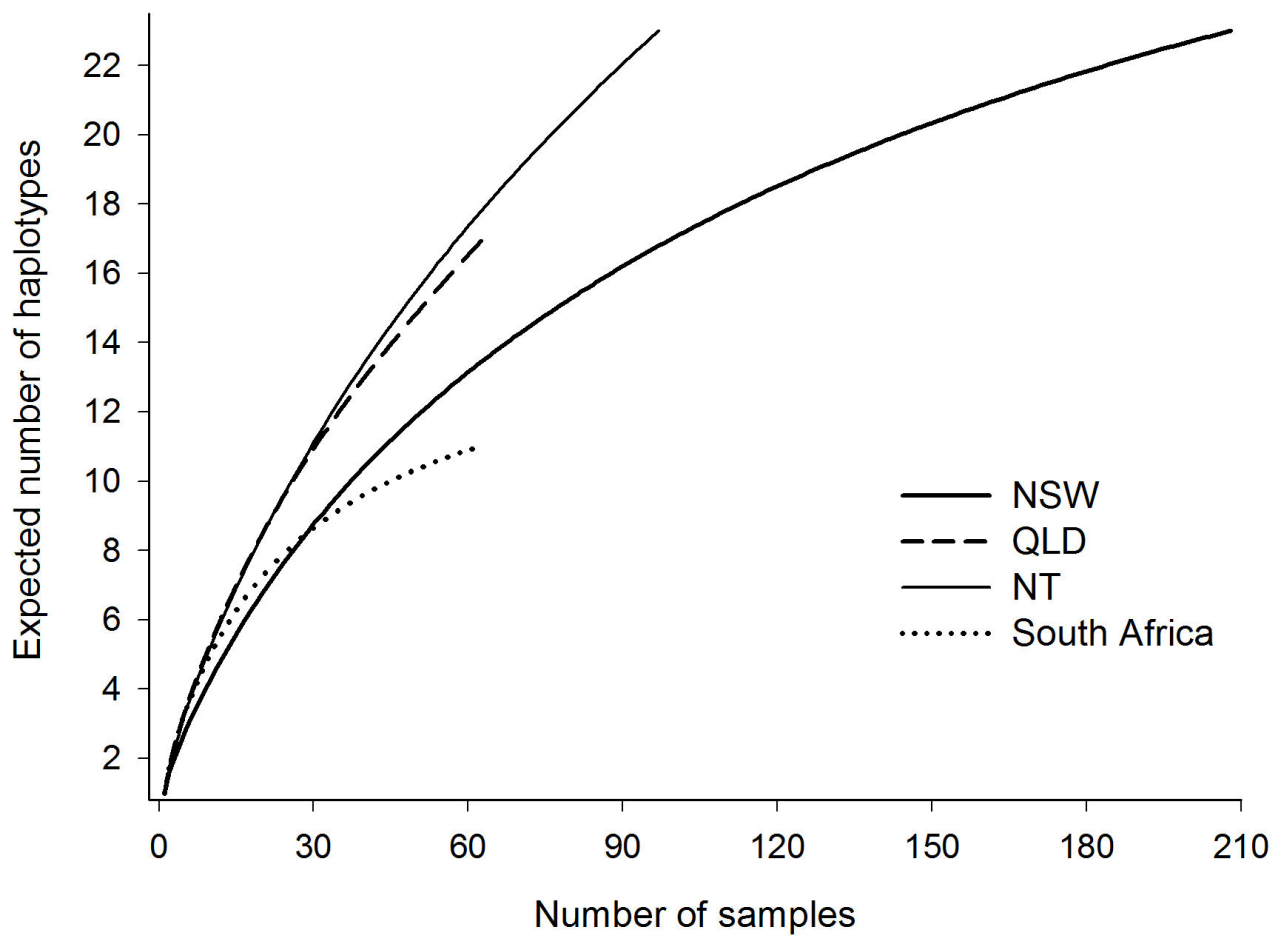
Rarefaction exact curves for sample locations.

Rarefaction exact curves indicated trends towards asymptotic relationships for both the NSW and South African locations (Figure 4), despite markedly different sample sizes. This suggests that the majority of the haplotypic diversities available at these two locations were most likely sampled. Steeper slopes were observed from QLD and NT waters (Figure 4), suggestive that some proportion of the available genetic diversities remained unsampled. Optimum sample size for the adequate representation of levels of genetic variation present in a given *C* *. brevipinna* population appears to be site dependent.

### Population genetic structure

**Figure 5:**
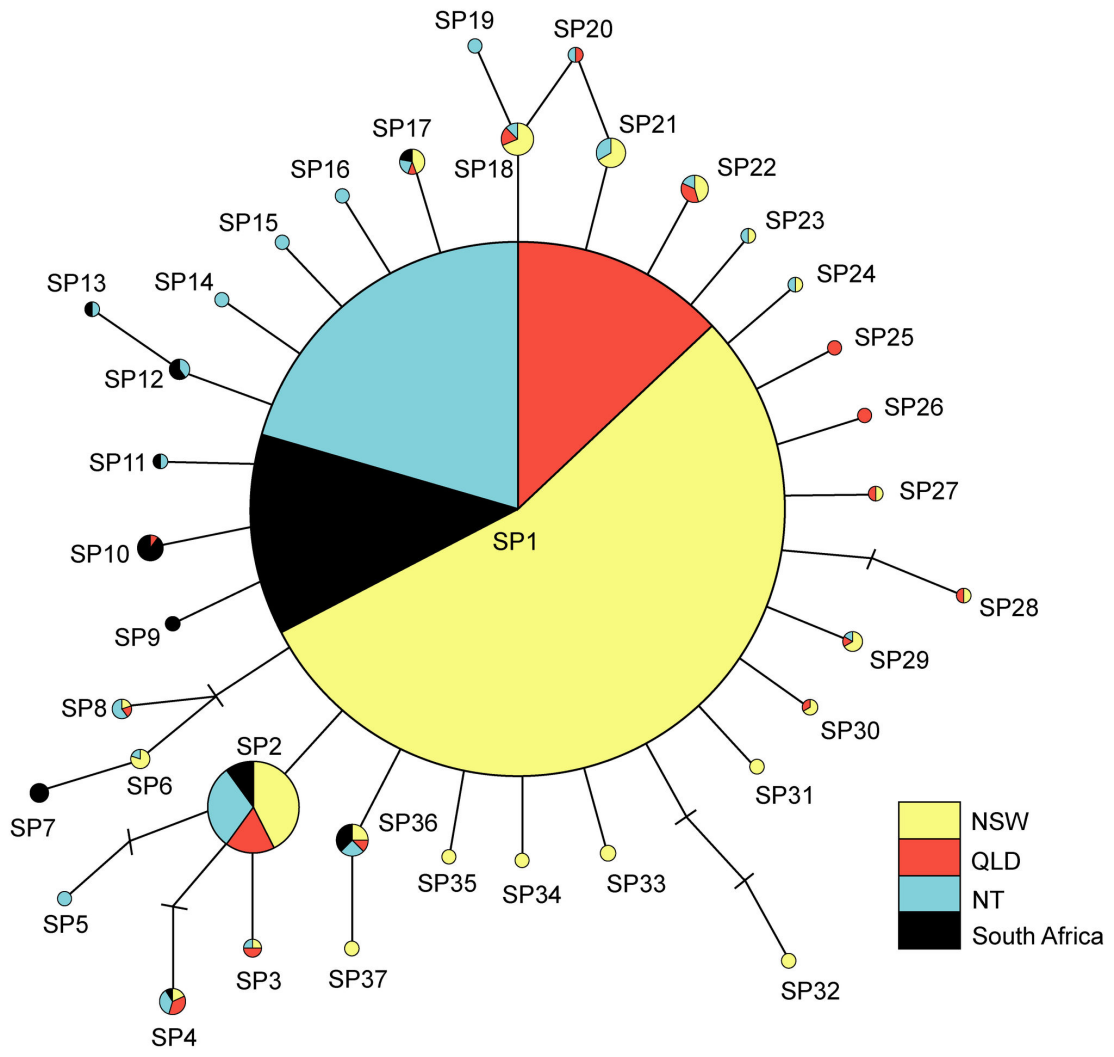
*Carcharhinus* *brevipinna* mitochondrial DNA ND4 haplotype network. Sizes of circles correspond to the number of individuals displaying each haplotype. Shading indicates the proportion observed from each of the four putative populations. (−) = mutational steps/missing haplotypes.

The haplotype network incorporating the four putative populations was shallow and shaped in a distinct ‘star-burst’ pattern, characterised by one central haplotype (SP1) surrounded by an array of low, or lower, frequency variants (SP2-SP27) (Figure 5). A high degree of haplotype sharing was observed among the four geographically-distinct populations, with the dominant haplotype (SP1) being common at each of the four locations and ~58% (or *n* = 21 of *n* = 36) of lower frequency haplotypes being shared between two or more locations (Figure 5, Table 2).

Despite this, AMOVA fixation indices detected significant levels of genetic differentiation between the four putative populations for both *F*-statistic metrics (Φ_ST_ = 0.01634, *p* = 0.0001; *F*_ST_ = 0.01493, *p* < 0.0035) (Table 4). We therefore reject the null hypothesis that *C* *. brevipinna* are panmictic in Australian and South African waters. Pairwise results, however, revealed some differences between the two measures of divergence. The Φ_ST_ metric detected genetic subdivision between South Africa and all Australian locations (pairwise Φ_ST_, range 0.02714–0.03508; *p* value, range 0.0000–0.0013), with all three comparisons significant after Bonferroni correction (Table 5). Φ_ST_ also detected genetic differentiation, albeit weaker, between NSW and QLD waters (pairwise Φ_ST_ = 0.01328, *p* < 0.016) which was also significant after sequential Bonferroni adjustment, as well as some evidence for genetic subdivision between NSW and NT (pairwise Φ_ST_ = 0.00669) which was significant at *p* < 0.05 but not after Bonferroni correction (Table 5). In contrast, the haplotype-frequency based analysis indicated significant genetic differentiation between the NSW and South African locations only (pairwise *F*_ST_ = 0.04056, *p* = 0.0008) (Table 5). All other pairwise *F*_ST_ comparisons, with the exception of QLD vs NT, were only marginally non-significant (pairwise *p* value, range 0.0510–0.0845). The finding of genetic homogeneity between QLD and NT was concordant between both *F*-statistics. A strong positive relationship, with high goodness-of-fit (*r* ^2^ = 0.86), was observed between pairwise genetic and geographic distances for *C* *. brevipinna* . This relationship, being driven entirely by differences between Australian locations and South Africa, was not statistically supported by a mantel test (*p* = 0.091).

**Table 4:** AMOVA analyses of spatial genetic variation for *Carcharhinus* *brevipinna* from Australian and South African waters.

Source of variation	d.f.	Test statistic	Sum of squares	Variance components	Percentage of variation (%)
Among populations	3	Φ_ST_	4.304	0.00916	1.63
	3	*F*_ST_	2.475	0.00508	1.49
Within populations	426	Φ_ST_	234.819	0.55122	98.37
	426	*F*_ST_	142.742	0.33507	98.51
Fixation indices		Φ_ST_ = 0.01634; *p* = 0.00010 (±0.00007)			
		*F*_ST_ = 0.01493; *p* = 0.00345 (±0.00041)			

**Table 5:** Comparison of pairwise *F*-statistic values between putative populations.

	NSW (*n* = 208)	QLD (*n* = 63)	NT (*n* = 97)	South Africa (*n* = 62)
NSW		0.01151 (0.0601)	0.00921 (0.0531)	***0.04056*** (0.0008)
QLD	***0.01328*** (0.0151)		−0.00704 (0.9099)	0.01306 (0.0845)
NT	0.00669* (0.0387)	−0.00507 (0.8166)		0.01411 (0.0510)
South Africa	***0.03494*** (0.0000)	***0.03508*** (0.0009)	***0.02717*** (0.0013)	

Observed Φ_ST_ values are below the diagonal, and *F*_ST_ values are above diagonal, with *p* values in parentheses. Bold italics indicate values significant after sequential Bonferroni correction (initial α = 0.0083). * Statistically significant at *p* ≤ 0.05, but not following Bonferroni adjustment.

**Figure 6:**
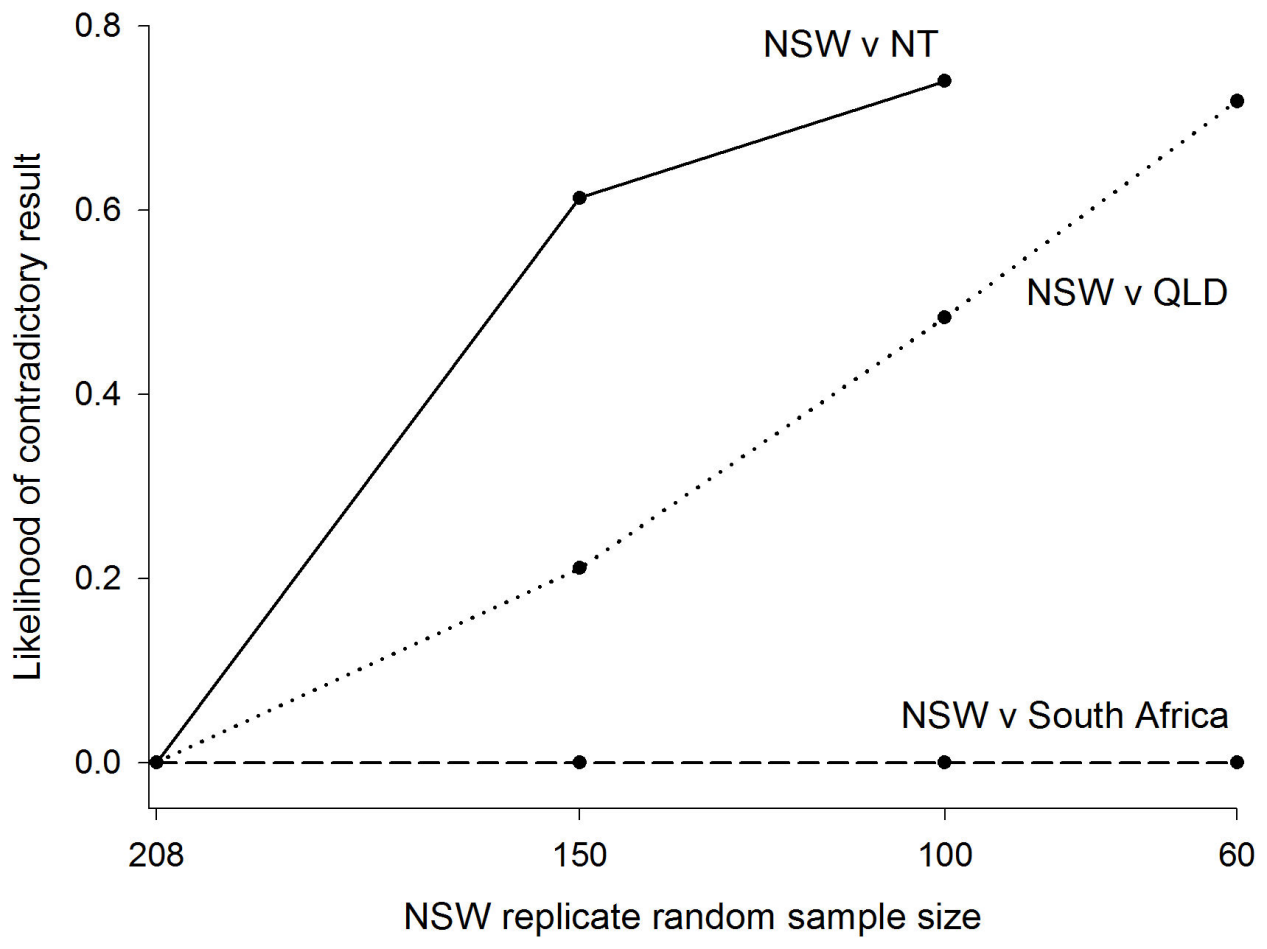
Likelihood of pairwise result contradicting that of the original analysis. Likelihoods computed based on 10,000 replicate random re-samples of the NSW population at varying sample sizes. Y-intercept represents original NSW population (*n* = 208).

**Figure 7:**
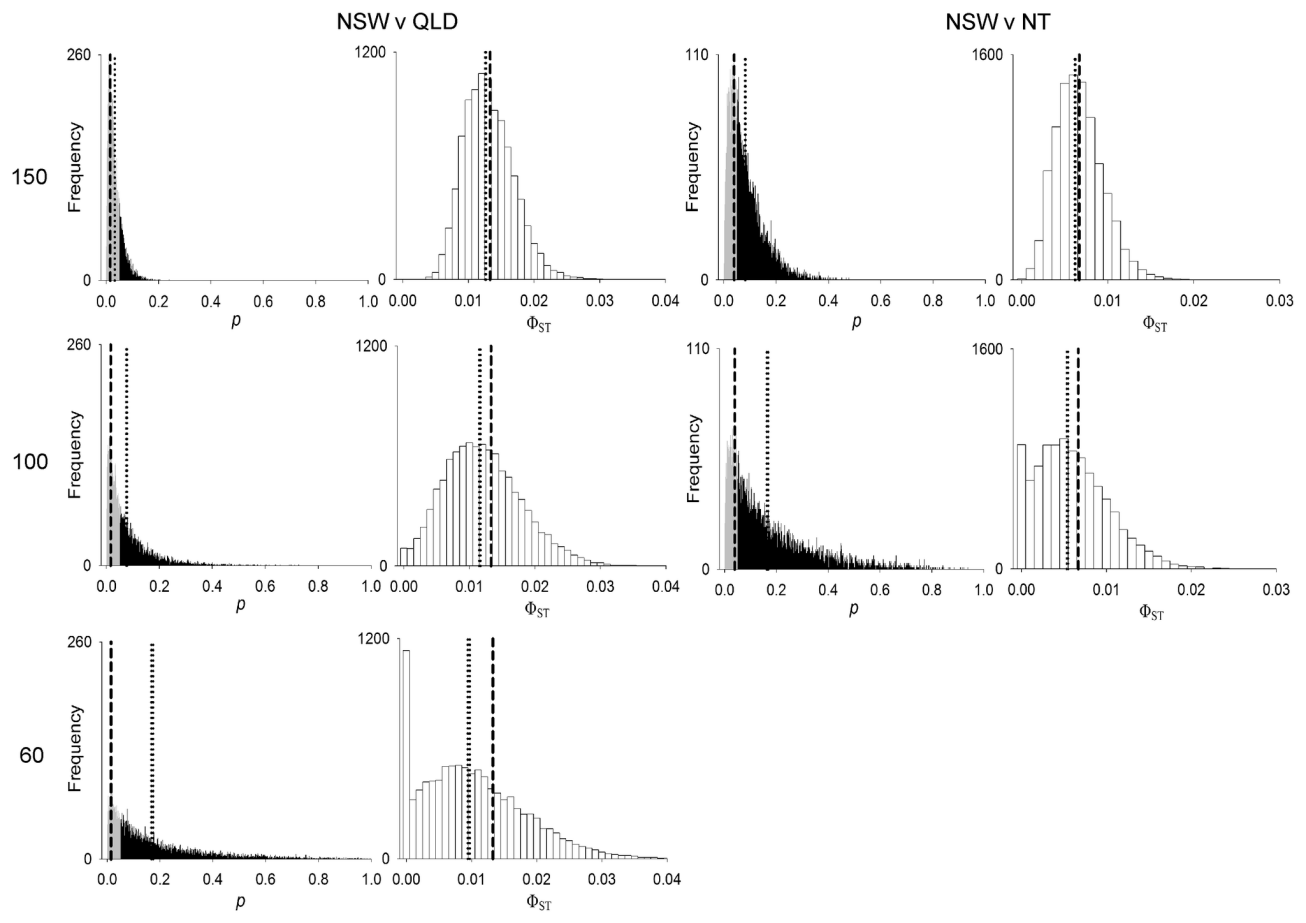
Pairwise Φ_st_ and *p* value distributions following random re-sampling simulations. NSW versus QLD and NT pairwise distributions based on 10,000 replicate random re-samples of the NSW population at *n* = 150, 100 and 60. Grey and black zones on *p* value distributions represent *p* ≤ 0.05 and *p* > 0.05 respectively. Dotted lines denote upper and lower 95% confidence intervals around sample means. Dashed lines indicate pairwise Φ_ST_ and *p* values generated by the original analysis.

Simulation was used to test the effect of a bias in the numbers of *C* *. brevipinna* sampled from NSW on the *F*-statistics analysis of pairwise population comparisons. Random re-samplings demonstrated an increasing likelihood of finding a non-significant result between NSW and QLD, and between NSW and NT, with decreasing NSW sample size (Figure 6). More specifically, for NSW vs QLD, 21.08% of replicate pairwise comparisons where sample size was set to 150 for NSW (and left at 63 for QLD) did not provide statistical support for the original analysis, for which sample size was 208 for NSW and 63 for QLD. This increased to 48.29% and 71.8% as the NSW sample size was reduced further to 100 and 60, respectively. Considering NSW vs NT, the likelihood of producing a contradictory result to that of the original analysis was high as NSW sample size was reduced. Where sample size was set to 150 for NSW (and left at 97 for NT), 61.32% of replicate pairwise comparisons did not provide statistical support for the original analysis, for which sample size was 208 for NSW and 97 for NT. This increased to 74% when the NSW sample size was reduced to 100. Further illustrating this point, as NSW sample size was reduced, pairwise Φ_ST_ and *p* value distributions revealed increasing variability in conjunction with decreasing mean Φ_ST_ and increasing mean *p* value relative to the output of the original analysis (Figure 7). This pattern was observed for both sets of locations. In contrast, replicate pairwise comparisons between NSW and South Africa displayed an unchanging, and zero percent, likelihood of generating a different result to that of the original analysis as NSW sample size was altered (Figure 6).

## Discussion and Conclusions

### Observer identification accuracy in an east Australian shark fishery

Observer accuracy was high (98.4%) in the identification of *C* *. brevipinna* in the NSW OTLF. This estimate is comparable to other target species within this same fishery; *C* *. obscurus* and *C* *. plumbeus* were correctly identified by fishery observers to accuracies of 96.6% and 99.4%, respectively (PT Geraghty, unpublished data). Given the fundamental importance of accurate catch-composition data in fisheries (and species) management [55,92,93], this high level of accuracy in the recognition of the three most harvested shark species (by number) in the NSW OTLF [53] confirms the usefulness of fishery-observer data in the management of this eastern Australian large shark fishery.

Our measure of observer accuracy (98.4%) for *C* *. brevipinna* in the NSW OTLF was higher than that reported for the same species by Tillett et al. [55] in the Northern Territory Offshore Net and Line Fishery (NT ONLF), for which observer accuracy was estimated at 87.2%. Higher identification accuracy in the NSW OTLF compared to the NT ONLF was not unexpected for this particular species given the difference in size class targeted by the two fisheries. The vast majority of the landed shark catch in the NSW OTLF is in the form of mature, adult individuals [53]. In contrast, the NT ONLF targets predominantly neonate and small juvenile life stages, illustrated by the fact that all sharks identified as *C* *. brevipinna* by observers in the latter fishery were ≤ 1.2m TL [55]. Size-at-capture is important as *C* *. brevipinna* is characterised by diagnostic traits that become increasingly discernible as an individual grows larger, most notably tooth shape and fin pigmentation [17]. At small sizes, *C* *. brevipinna* can be difficult to distinguish from a range of other morphologically-similar carcharhinid species [17].

### Evolutionary history in the southern Indo-Pacific

The *C* *. brevipinna* haplotype network was distinctly star-shaped, characterised by a single dominant haplotype surrounded by a high number of low, or lower, frequency variants. This central, and presumably ancestral, haplotype was prominent in all three Australian sample locations, as well as off the coast of South Africa - evidence that Australian and South African waters share common ancestry in this species.

The pattern of genetic diversity observed here in *C* *. brevipinna* is indicative of a contemporary demographic expansion event having occurred throughout the southern Indo-Pacific. This hypothesis is supported by a range of evidence: the distinctly ‘star-burst’ haplotype network denoted by numerous low-frequency mutations, mismatch distributions and neutrality test statistics suggesting strong departures from mutation-drift equilibrium for all four putative populations and the observed combination of generally high haplotype and low nucleotide diversities [82,83,94-96]. Attempts at dating this population expansion event were abandoned in the absence of mutation-rate estimates for ND4 in elasmobranchs.

It must be noted here, however, that spatial sample coverage in the present study is limited to only a very small area of this species’ global distribution range, which includes much of the world’s tropical and warm-temperate continental shelf waters [17]. Therefore, in the absence of genetic analysis of samples representative of the entire distribution of the species, we are unable to determine whether or not this rapid population growth was a worldwide event or was restricted to the southern Indo-Pacific.

The strong signal of population expansion reported here in *C* *. brevipinna* is unprecedented among sharks, with comparable signals more commonly associated with taxa such as humans [2] and teleost fishes [6,97,98]. Evidence for population expansion has, however, been presented for some shark species through analyses of mismatch distributions [8,99], star-like haplotype networks [40,100,101], or combinations of the latter two supported by neutrality indices [102,103].

### Contemporary genetic structuring

This study marks the first dedicated assessment of genetic structure in *C* *. brevipinna* . The application of two metrics of genetic divergence (Φ_ST_ and *F*_ST_) demonstrated that population genetic findings can be dependent on the *F*-statistic employed - especially pertinent where subdivision is at the margins of statistical significance [98]. We therefore encourage the concurrent use of both metrics as standard practice in population genetic studies.

With this in mind, genetic differentiation was detected over a broad spatial scale between Australian and South African waters. This finding based on mtDNA was not unexpected and, being consistent with a range of other shark population genetic studies [35-37,99,104-107], re-iterates that large oceanic expanses (in this case the Indian Ocean) represent robust barriers to contemporary gene flow in coastal shark species.

Evidence for genetic subdivision, albeit weak, was also detected over finer spatial scales within Australian waters, i.e. between NSW and both QLD and, to a lesser degree, NT. Genetic homogeneity was observed between QLD and NT waters. These results tentatively suggest that gene flow is restricted to some degree along Australia’s eastern continental margin as well as between the south-eastern and northern coastlines, and that gene flow is unencumbered between north and north-eastern Australian waters. These findings were somewhat unexpected given *C* *. brevipinna* ’s potential for active dispersal. That said, however, genetic differentiation has previously been detected in similar and related shark species over comparable geographic scales in Australian waters [31,42,108,109], as well as those of the Gulf of Mexico and north-western Atlantic [12,30].

Reproductive philopatry, or the fidelity of gravid females to nursery areas, is typically invoked to explain fine-scale genetic structuring (based on maternally-inherited mtDNA) in the absence of barriers to dispersal for highly-vagile sharks [12,30,31,34,41,109]. Confidently discerning this sex-biased behavioural trait, however, is complex and relies on a robust experimental design involving the exclusive sampling of neonates, or adult females at time of parturition rather than during dispersal, from spatially discrete areas [12,32]. The collection of tissues in the present study was generally reliant on both spatial and temporal opportunistic sampling, rather than according to a dedicated experimental design. Nevertheless, tissues from NT and QLD were almost exclusively sampled from neonates and small juveniles, with length-frequency modes at 90 cm and 95–100 cm TL respectively (Figure 2). While it is conceivable that the fine-scale genetic structuring observed in this study reflects signs of reproductive philopatry, the only meaningful test of this hypothesis would be a comparison of the NT and QLD locations between which our data failed to detect genetic differentiation.

Consideration of our results in light of those by Ovenden et al. [40], however, would suggest that an affinity for nearshore habitat for nursery purposes in *C* *. brevipinna* has influenced our findings of fine-scale genetic differentiation to some degree. In their study, Ovenden et al. [40] failed to detect evidence for genetic subdivision along Australia’s east coast in milk sharks ( *Rhizoprionodon* *acutus* ) using ND4 sequence data. *Rhizoprionodon* *acutus* , a considerably smaller-bodied and presumably less-vagile species than *C* *. brevipinna* , conforms to a population model characterised by permanent habitation of nearshore waters without the use of discrete nursery areas [110]. In contrast, the exclusive use of nearshore habitat by *C* *. brevipinna* for parturition and juvenile development is well documented (24, 56-59). Differing life-cycles denoted by varying usage of nearshore habitat, therefore, may account for these contrasting genetic structures observed along Australia’s east coast.

Alternatively, genetic differentiation between NSW and NT may be a relict signature of repeated periods of temporary isolation due to the rise and fall of the Torres Strait land bridge caused by fluctuating sea levels during the Pleistocene epoch [111,112]. This physical, yet temporary, barrier to movement (and hence gene flow) in marine taxa between the east coast and areas west of the Cape York Peninsula was hypothesised to account for contemporary genetic subdivision in pigeye sharks ( *Carcharhinus* *amboinensis* ) [42] which, like *C* *. brevipinna* , have a distribution restricted to northern regions in Australian waters [17]. Under this hypothesis, however, one would anticipate a similar level of genetic differentiation between QLD and NT, rather than genetic homogeneity as observed.

Similarly, a marked change in marine environment coinciding with the Tropic of Capricorn (Figure 1) represents an alternative hypothesis explaining restricted contemporary gene flow between south-eastern and more northern Australian waters [108]. This latitudinal line discretely separates the NSW population from both QLD and NT populations (with the exception of one individual from southern QLD waters), and delineates a shift from temperate and subtropical continental shelf waters, rocky coastline and drowned river valleys to a largely reef and lagoon-dominated tropical ecosystem.

### Project limitations

This study was subject to a range of limitations requiring careful consideration. To begin with, very low values for both Φ_ST_ and *F*_ST_ metrics (resulting from high incidence of haplotype sharing of both ancestral and recently derived haplotypes among all four putative populations, coupled with generally shallow divergence between mutational variants) is suggestive of a slow rate of mutation in the ND4 gene region. This raises considerable doubt as to the ability of ND4 to effectively discriminate population structure in *C* *. brevipinna* . For example, pairwise *F-*statistic estimates involving the South African population were demonstrably low in the present study (range, 0.01306–0.04056) compared to others reporting genetic differentiation in sharks over comparable spatial scales (range, 0.18–0.991) (Table 6). Given that these previous studies were all based on analysis of a different mitochondrial locus (i.e. the control region), a slower rate of mutation in the ND4 region may account for the comparatively low *F*-statistics observed here. However, a hypothesis based on low ND4 mutation rate is challenged by the findings of both Dudgeon et al. [113] and Ovenden et al. [114] who demonstrated that for *C* *. limbatus* , Australian blacktip ( *Carcharhinus* *tilstoni* ) and zebra ( *Stegostoma* *fasciatum* ) sharks, ND4 was the most polymorphic of a range of mtDNA markers, including the control region. Alternatively, therefore, low *F*-statistic values associated with observed genetic structuring between Australia and South Africa, as well as within Australian waters, may reflect continued low-level gene flow, or a recent cessation of gene exchange, between subdivided locations. Until the relative mutation rates of ND4 and CR are determined for *C* *. brevipinna* , however, or this study is reassessed via sequencing of CR, it is impossible to confidently support or refute the abovementioned hypotheses. Moreover, this issue emphasises the limitations inherent in the analysis of only one mtDNA locus.

**Table 6:** Mitochondrial divergence metrics for population pairwise comparisons involving Australia and South Africa.

Pairwise comparison	Species	Gene	*F*_ST_	Φ_ST_	Reference
AUS v SA	*Carcharhinus* *brachyurus*	CR		0.97	[36]
	*Carcharhinus* *obscurus*	CR		0.18	[37]
	*Carcharodon* *carcharias*	CR	0.81		[104]
	*Carcharhinus* *brevipinna*	ND4		0.03216	Present study
EAUS v SA	*Carcharias* *taurus*	CR	0.813		[105]
	*Carcharhinus* *brevipinna*	ND4	0.04056	0.03494	Present study
NEAUS v SA	*Carcharhinus* *plumbeus*	CR		0.588	[35]
	*Carcharhinus* *brevipinna*	ND4	0.01306	0.03508	Present study
WAUS v SA	*Carcharias* *taurus*	CR	0.676		[105]
	*Carcharhinus* *plumbeus*	CR		0.6165	[35]
	*Sphyrna* *lewini*	CR		0.991	[99]
	*Sphyrna* *lewini*	CR	0.45		[107]
SAUS v SA	*Galeorhinus* *galeus*	CR		0.34	[106]

CR = control region, ND4 = NADH dehydrogenase subunit 4.

AUS = Australia (general), EAUS = eastern Australia, NEAUS = north-eastern Australia, WAUS = western Australia, SAUS = southern Australia, SA = South Africa

The clear bias in sample sizes weighted towards the NSW population represents another major limitation of this study. Random-resampling simulations provided some evidence that the detections of significant genetic differentiation within Australian waters (i.e. between NSW and QLD, and NSW and NT) were driven in large part by this bias. Replicate pairwise comparisons for both sets of locations indicated an increasing likelihood of finding a non-significant result as the NSW sample size decreased towards a more balanced analysis. This either serves to emphasise the weak nature of the observed fine-scale genetic subdivisions within Australian waters, or draw their actual existence into question. Conversely, replicate pairwise comparison between NSW and South Africa returned a significant difference independent of the NSW sample size, hence reinforcing the strength of the genetic subdivision between the latter two regions, and indicating that the original analysis was robust to the bias in sample size in this instance.

Rarefaction analysis added further uncertainty regarding the reliability of our fine-scale findings reported in the present study. NSW and South Africa were the only two locations at which adequate levels of the available genetic diversities were likely sampled, hence confirming the robustness of the comparison between these two putative populations. In contrast, a proportion of the available diversity appeared to have remained unsampled from QLD and NT, suggesting that findings emanating from comparisons involving the latter two locations should be treated with some degree of caution. Rarefaction curves demonstrated that the optimum sample size required to accurately represent levels of haplotypic variation, and in turn to confidently discern haplotype relative frequencies, within any given *C* *. brevipinna* population is site dependent. For Australian locations, sample sizes in excess of 100 were required for robust comparisons, whereas a sample size of ~60 appeared sufficient for South African waters.

### Implications for management and future direction

The generally high genetic diversity reported here in *C* *. brevipinna* is cause for optimism when considering the management and conservation of this commercially-targeted species in southern Indo-Pacific waters. *Carcharhinus* *brevipinna* exhibited high haplotype numbers and similar or high haplotypic diversity (*n*_*H*_ = 23, *h* = 0.5984, *n* = 208) compared to *C* *. obscurus* (*n*_*H*_ = 12, *h* = 0.5224, *n* = 301) and *C* *. plumbeus* (*n*_*H*_ = 11, *h* = 0.2826, *n* = 440), two closely-related species, off Australia’s east coast (PT Geraghty, unpublished data). Comparatively high haplotype numbers implies that *C* *. brevipinna* may display a greater resilience to a loss of genetic diversity, as a result of high-intensity fishing pressure, than these other commercially-targeted shark species in Australian waters.

The lower genetic diversity observed in *C* *. brevipinna* from the south-eastern zone (*h* = 0.5984), compared to QLD (*h* = 0.7424) and NT (*h* = 0.7279), may be accounted for by NSW representing sampling of the species’ southern-most distribution limit [17]. Range limits are associated with extreme and/or unstable environmental conditions, and have been hypothesised to result in low population density, increased genetic drift and inbreeding and, consequently, lower genetic diversity [115,116]. Alternatively, lower genetic diversity in NSW may be a consequence of greater harvest pressure in the region. This hypothesis, however, is difficult to support given the absence of robust data permitting a direct comparison of historical harvest levels of *C* *. brevipinna* between NSW, QLD and NT, as well as a lack of knowledge pertaining to original population sizes and periods of time required to affect quantifiable reductions in genetic diversity.

Our genetic structure results indicate the delineation of two management units for *C* *. brevipinna* in the southern Indo-Pacific – Australia and South Africa. The most appropriate boundary between these management units, however, is unknown and would require more detailed spatial sampling within the Indian Ocean basin. Our data also suggest, albeit tentatively, two management units within Australian waters – south-eastern (NSW) and northern (QLD and NT) Australia. This implies that, in the event of a population collapse in south-eastern Australia, recovery of genetic diversity would rely largely on reproduction by surviving local individuals in NSW waters. Currently, each Australian state is independently responsible for the management of shark fishing operations occurring within its respective waters, with little to no collaboration across jurisdictional borders. Our results suggest that the independent management of NSW and QLD *C* *. brevipinna* populations is perhaps appropriate, but that cooperation between QLD and NT would be prudent.

In light of the limitations of the present study, however, we recommend this work be considered as a starting point for evaluations of genetic structure in this commercially-important species, rather than a study upon which definitive management decisions are made. Moreover, we strongly urge future studies to focus on achieving greater population structure resolution via more extensive sampling within Australian waters, as well as throughout this species’ global distribution range, in conjunction with analysis of nuclear and/or additional mitochondrial markers. Such studies, conducted in association with active tagging and tracking, would assist with more robust allocations of management units, and hence the sustainable exploitation of this target species.

## Supporting Information

Table S1 **Polymorphic sites for mitochondrial DNA ND4 haplotypes defined for *Carcharhinus* *brevipinna* .**
(DOC)
