# Correction: Population Expansion and Genetic Structure in *Carcharhinus brevipinna* in the Southern Indo-Pacific

**DOI:** 10.1371/journal.pone.0094738

**Published:** 2014-04-09

**Authors:** The PLOS ONE Staff

The second affiliation for the fourth author is not indicated. Sabine P. Wintner is affiliated with KwaZulu-Natal Sharks Board, Umhlanga Rocks, KwaZulu-Natal, South Africa and Biomedical Resource Unit, University of KwaZulu-Natal, Durban, South Africa.
